# The relationship between serum 25-hydroxyvitamin D levels and dyslipidemia risk: insights from a study of elderly Chinese diabetic foot ulcer patients

**DOI:** 10.3389/fendo.2025.1699243

**Published:** 2025-12-02

**Authors:** Chuyi Hong, Huiming Qi, Xing Gao

**Affiliations:** Department of Rehabilitation, People’s Liberation Army Air Force Special Medical Center, Beijing, China

**Keywords:** 25-hydroxy vitamin D, dyslipidemia, diabetic foot ulcer, Chinese, elderly

## Abstract

**Objective:**

Existing evidence links low serum vitamin D concentrations to diabetic foot ulcer (DFU) development, but evidence on the interaction between vitamin D and blood lipids—especially in the elderly—remains limited. This study systematically evaluated the association between serum 25-hydroxyvitamin D [25(OH)D] levels and dyslipidemia in elderly DFU patients, and explored potential non-linear dose-response patterns and interaction effects.

**Methods:**

A total of 181 elderly (≥60 years) DFU patients hospitalized at the Characteristic Medical Center of Air Force Medical University in China from January 1, 2020 to May 31, 2024 were retrospectively included. Serum 25(OH)D and biochemical markers were measured using standardized laboratory protocols. (1) Differences in the detection rate of dyslipidemia were analyzed using 25(OH)D categorical variables to screen target lipid variables; (2) Multivariate logistic regression was used to assess the association between serum vitamin D and abnormal target lipid variables; (3) Restricted cubic spline (RCS) and curve fitting were employed to evaluate non-linear dose-response trends, and further piecewise regression was conducted to examine potential threshold effects; (4) Subgroup analyses were performed to investigate interactions among age, gender, duration of hyperglycemia, lifestyle, and comorbidities.

**Results:**

The 25(OH)D ≥50nmol/L group had a significantly lower abnormal triglyceride (TG) rate than the other two groups (*p* = 0.015). Logistic regression showed a dose-dependent negative correlation between 25(OH)D and abnormal TG; after covariate adjustment, each 1nmol/L increase in 25(OH)D reduced abnormal TG risk by 3.3% (adjusted OR = 0.967, 95%CI: 0.943–0.991, *p* = 0.009). RCS analysis confirmed an approximate linear negative correlation, with stronger protection at 25(OH)D >30.322nmol/L and no significant non-linearity. Threshold analysis identified 43.48nmol/L as the inflection point (OR = 0.907, *p* = 0.043 above this value), though model fitting improvement was unclear. Subgroup analyses showed significant protective effects of 25(OH)D except in season-, Wagner grade-stratified groups and those with coronary heart disease, with no significant interactions.

**Conclusion:**

In Chinese elderly DFU patients aged ≥60 years, serum vitamin D levels are dose-dependently negatively correlated with the risk of abnormal TG. However, this retrospective observational study only identifies an association, not causality, due to observational design limitations. Nevertheless, assessing elderly DFU patients’ vitamin D status may aid management, requiring verification via prospective studies or RCTs.

## Introduction

Diabetes mellitus (DM) is a global public health challenge, and its complications impose a heavy burden on individuals and healthcare systems (1). According to data from the International Diabetes Federation (IDF), over 500 million people worldwide are living with diabetes, and this number is projected to exceed 700 million by 2045 (2). Diabetes-related foot disease encompasses one or more of the following conditions affecting the feet of patients with current or previously diagnosed diabetes: peripheral neuropathy, peripheral arterial disease (PAD), infection, ulceration, neuroosteoarthropathy, gangrene, or amputation. Foot ulcers, as one of the most severe complications of diabetes, are a major cause of reduced quality of life and substantial economic costs for affected individuals (3).

Statistical data indicate that approximately 34% of patients with diabetes will develop diabetic foot ulcers(DFU) (4). Around 10% of DF patients die within 1 year after ulcer onset (5), and the 5-year mortality rate of patients with DFUs can reach 50–70%, which is higher than that of many types of cancer (6).

DFU arises from multiple pathogenic factors, including vascular lesions, neuropathy, and microenvironmental disturbances such as tissue ischemia, hypoxia, and hyperglycemia. These microenvironmental disturbances disrupt the progression of the wound healing process, leading to delayed or non-healing wounds and triggering a series of clinical complications (7). In the elderly population, these mechanisms are further complicated by age-related decline in tissue repair capacity, comorbidities such as hypertension and cardiovascular diseases, and polypharmacy, rendering the management of DFU extremely challenging (8). This underscores the need to deepen the understanding of modifiable factors that influence DFU progression and associated metabolic disorders.

Vitamin D has long been recognized for its crucial role in calcium homeostasis, and it has now been confirmed as a key player in immune regulation, anti-inflammation, and cell differentiation—functions that are essential for wound healing and vascular health (9). A growing body of evidence suggests that vitamin D deficiency is associated with an increased risk of DFU development and delayed wound healing (10). Notably, recent research (11) further reveals that genetic variations in the vitamin D receptor (VDR) pathway can modulate DFU risk: specific VDR single nucleotide polymorphisms (SNPs) may amplify the susceptibility to DFU in individuals with vitamin D deficiency. It can thus be hypothesized that vitamin D deficiency itself is already associated with diabetic foot ulcers (DFU), and that VDR genetic variations further exacerbate this risk. Consequently, investigating vitamin D levels in this population (as well as their interactions with lipid metabolism) holds greater clinical relevance. Mechanistically, 25(OH)D deficiency may impair macrophage function, reduce the production of antimicrobial peptides, and interfere with angiogenesis—all of which can hinder ulcer healing (9).However, despite the increasing attention to the association between vitamin D and the pathogenesis of DFU, its interaction with lipid metabolism in this context remains understudied, especially in elderly patients with DFU.

Dyslipidemia in diabetes is characterized by elevated fasting and postprandial triglycerides (TG), low high-density lipoprotein cholesterol (HDL-C), and elevated low-density lipoprotein cholesterol (LDL-C). These lipid changes represent the primary link between diabetes and increased cardiovascular risk in patients with diabetes (12).

In previous studies, remnant cholesterol and triglycerides were independently associated with an increased risk of diabetic foot disease in patients with type 1 diabetes (T1D) (13); meanwhile, standard lipid parameters, particularly TG and HDL-C levels, may help assess the risk of developing DFU (14).

Current evidence indicates that vitamin D supplementation can significantly promote DFU healing by reducing blood glucose levels, alleviating inflammation, and mitigating oxidative stress (15). However, these findings are primarily derived from patients with uncomplicated diabetes or healthy populations; data specifically targeting elderly patients with DFU remain scarce, creating a critical gap in our understanding of whether vitamin D influences lipid abnormalities in this high-risk subgroup.

Furthermore, the nature of the relationship between 25(OH)D and dyslipidemia—whether linear, dose-dependent, or threshold-dependent—remains unclear. Clarifying these patterns could inform targeted vitamin D supplementation strategies. For instance, if a threshold effect exists (e.g., lipid benefits plateau above a specific 25(OH)D level), it would help guide personalized interventions to optimize therapeutic outcomes. Additionally, potential interactions involving demographic factors (age, gender), lifestyle factors (smoking, alcohol consumption), or comorbidities (duration of hyperglycemia, cardiovascular disease) have not been systematically evaluated in elderly patients with DFU, which limits the generalizability of existing research.

To address these gaps, this study aims to systematically investigate the association between serum 25(OH)D levels and dyslipidemia in elderly patients with DFU (≥60 years old). Specifically, we seek to (1): analyze differences in the prevalence of lipid abnormalities across different 25(OH)D strata; (2) quantify the relationship between 25(OH)D and target lipid parameters using multivariate logistic regression; (3) explore non-linear dose-response patterns and potential threshold effects via restricted cubic spline (RCS) and piecewise regression; and (4) assess the effect modification of key demographic and clinical factors. By elucidating these associations, our findings may help refine comprehensive management strategies for elderly patients with DFU, highlighting the optimization of vitamin D status as a potential adjunct to conventional treatments (e.g., blood glucose control, wound care, infection management) to reduce lipid-related cardiovascular risk and improve overall prognosis.

## Methods

### Study population and data collection

This study included patients with DFU aged ≥60 years who were hospitalized at the Characteristic Medical Center of Air Force Medical University between January 1, 2020, and May 31, 2024. For this analysis, participants were excluded if they were aged <60 years or had no measured serum vitamin D_3_ levels. Ultimately, the final study population comprised 181 elderly patients, who underwent physical examinations and blood sample collection upon admission. Data collection was conducted by a team of professional physicians.

### Data collection

Inclusion criteria: (1)Meeting the clinical diagnostic criteria for DFU; (2) Aged ≥60 years, regardless of gender.

Exclusion criteria: (1)Patients with severe acute diabetic complications (e.g., diabetic ketoacidosis or hyperosmolar nonketotic coma); (2)Patients with severe hepatic or renal insufficiency (e.g., decompensated cirrhosis or end-stage renal disease); (3) Patients with a past or current diagnosis of malignant tumors that may affect DFU prognosis; (4) Patients with incomplete clinical data or those who discontinued treatment during the intervention period. Individuals who met the above DFU criteria were included in the study.

Clinical medical histories were collected to capture participants’ long-term exposures and background characteristics. Smoking and alcohol consumption statuses were categorized as “yes” or “no” based on self-reported current or previous tobacco use and alcohol intake. Hypertension was defined as a systolic blood pressure ≥140 mmHg, diastolic blood pressure ≥90 mmHg, or current use of antihypertensive medications.

### Measured indicators and laboratory measurements

Collected lipid indicators included total cholesterol (TC), triglycerides (TG), high-density lipoprotein cholesterol (HDL-C), low-density lipoprotein cholesterol (LDL-C), and non-high-density lipoprotein cholesterol (non-HDL-C). The non-HDL-C value was calculated as TC value minus HDL-C value (16).

Venous blood was collected after a 12-hour fast. The glucose oxidase method was used to measure fasting plasma glucose, and plasma glycated hemoglobin A1c (HbA1c) was detected by high-performance liquid chromatography combined with affinity chromatography (Premier HB9210, Trinity Biotech, Kansas, USA). Serum 25(OH)D was detected using the enzyme-linked immunosorbent assay (ELISA).Lipid indicators were determined using an automatic biochemical analyzer.

All detections strictly followed laboratory standard operating procedures, and regular calibration was performed using national reference materials to ensure the accuracy and reliability of the detection results. This study protocol was approved by the Ethics Committee of the Characteristic Medical Center of the Chinese People’s Liberation Army Air Force (approval number: 2024-26-PJ01).

### Definition of outcomes

The primary outcome of this study was the presence of dyslipidemia at hospital admission. The stratification criteria for dyslipidemia were based on the Chinese Guidelines for the Management of Dyslipidemia in Adults (2016 Revised Edition) (16). Specifically, for participants, total cholesterol (TC) elevation was defined as TC ≥ 5.2 mmol/L; triglyceride (TG) elevation was defined as TG ≥ 1.7 mmol/L; high-density lipoprotein cholesterol (HDL-C) reduction was defined as HDL-C ≤ 1.0 mmol/L; low-density lipoprotein cholesterol (LDL-C) elevation was defined as LDL-C ≥ 3.4 mmol/L; and non-high-density lipoprotein cholesterol (non-HDL-C) elevation was defined as non-HDL-C ≥ 4.1 mmol/L.

### Statistical analysis

Characterization of Exposure Variable: 25(OH)D levels were expressed in two forms: categorical and continuous variables. The categorical variable was divided into three groups based on the 25(OH)D levels of the study population: <30 nmol/L, 30–50 nmol/L, and ≥50 nmol/L.

Baseline characteristics were summarized using mean ± standard deviation for continuous variables and frequency (percentage) for categorical variables. Independent samples t-test and chi-square test were used to assess group comparisons between patients with and without abnormal triglycerides (TG), respectively.

First, to evaluate the association between serum 25(OH)D levels and dyslipidemia, we initially performed chi-square tests to compare the detection rates of lipid metabolism abnormalities across different 25(OH)D groups.

Covariate Selection and Screening: Meaningful covariates were identified based on clinical relevance and statistical significance. The variance inflation factor (VIF) was used to assess multicollinearity, which informed the inclusion of covariates in the models.

Subsequently, multivariate logistic regression models were constructed with progressive adjustments: Model (1) Unadjusted; Model (2) Adjusted for variables with statistically significant differences among the three groups (as shown in **Table 1**), including age, gender, season of 25(OH)D measurement, duration of hyperglycemia, admission systolic blood pressure, and admission diastolic blood pressure.

**Table 1:** Main characteristics of patients included in this study.

Characteristic	<30nmol/L N=74	30~50nmol/L N=71	≥50nmol/L N=36	χ^2^/F/H value	*P* value
Age/year	71.09 ± 6.72	69.24 ± 5.75	68.73 ± 6.82	2.278	0.105
BMI/(kg/m^2^)	25.00 ± 3.44	24.46 ± 3.04	23.42 ± 2.93^a^	2.245	0.110
Gender, male	42(56.8)	49(69.0)	27(75.0)	4.185	0.125
Season				17.670	0.007
Spring	20(27.0)	15(21.1)	1(2.8)		
Summer	12(16.2)	19(26.8)	15(41.7)		
Autumn	22(29.7)	14(19.7)	13(36.1)		
Winter	20(27.0)	23(32.4)	7(19.4)		
Admission systolic blood pressure	143.27 ± 22.03	134.56 ± 21.65^a^	142.89 ± 21.02	3.359	0.037
Admission diastolic blood pressure	77.47 ± 10.29	73.43 ± 11.32^a^	80.72 ± 13.53	5.284	0.006
Diabetes-related risk factors					
HbA1c(%)	8.94 ± 1.83	8.77 ± 1.36	8.15 ± 1.55	0.763	0.470
Duration of hyperglycemia (year)	21.54 ± 9.20	20.43 ± 8.04	17.47 ± 10.46^a^	2.470	0.087
Classification of duration of hyperglycemia (year)				4.086	0.132
<20 years	33(44.6)	40(56.3)	23(63.9)		
≥20 years	41(55.4)	31(43.7)	13(36.1)		
Diabetic retinopathy	44(59.5)	36(50.7)	17(47.2)	1.857	0.398
Diabetic peripheral neuropathy	70(94.6)	66(93.0)	33(91.7)	0.559	0.799
Diabetic peripheral vascular disease	58(78.4)	58(81.7)	25(69.4)	2.101	0.341
Wagner classification				7.212	0.514
1	4(5.4)	11(15.5)	6(16.7)		
2	13(17.6)	12(16.9)	7(19.4)		
3	22(29.7)	17(23.9)	7(19.4)		
4	34(45.9)	31(43.7)	15(41.7)		
5	1(1.4)	0(0.0)	1(2.8)		
Other diagnoses					
Hypertension	55(74.3)	51(71.8)	22(61.1)	2.103	0.365
Anemia	33(44.6)	31(43.7)	15(57.7)	1.634	0.468
Coronary heart disease	22(29.7)	24(33.8)	6(16.7)	3.511	0.180
Lower extremity occlusive arteriosclerosis	38(51.4)	33(46.5)	19(52.8)	0.528	0.792
Lifestyle					
Current smoking	8(10.8)	14(19.7)	4(11.1)	2.520	0.293
Current drinking	14(18.9)	17(23.9)	6(16.7)	0.897	0.678
Blood lipid indices					
TC/(mmol/L)	3.81 ± 1.02	3.75 ± 1.05	3.42 ± 0.78^a^	2.021	0.136
TG/(mmol/L)	1.28(1.01, 1.92)	1.34(0.97, 2.27)	1.15(1.01, 1.42)	2.745	0.253
HDL-C/(mmol/L)	0.95 ± 0.28	0.94 ± 0.24	0.92 ± 0.28	0.195	0.823
LDL-C/(mmol/L)	2.15 ± 0.79	2.08 ± 0.78	1.94 ± 0.63	0.893	0.411
non-HDL-C/(mmol/L)	2.82 ± 1.07	2.81 ± 0.97	2.50 ± 0.74	1.540	0.217

Values are presented as mean ± standard deviation. ^a^*p* < 0.05: compared with the 25(OH)D <30nmol/L group. Season: Season of 25-hydroxyvitamin D test; TC: Total cholesterol; TG: Triglyceride; HDL-C: High-density lipoprotein cholesterol; LDL-C: Low-density lipoprotein cholesterol; non-HDL-C: Non-high-density lipoprotein cholesterol.

Next, to assess potential non-linear relationships between 25(OH)D levels and abnormal TG, restricted cubic spline (RCS) analysis, generalized additive model (GAM), and smooth curve fitting were conducted. The number of knots in the RCS models was determined using the Akaike Information Criterion (AIC) to balance model fit and complexity [16]. By comparing AIC values of models with different numbers of knots, the optimal number of knots for both Model 1 and Model 2 was set to 3. Piecewise regression was implemented using the “segmented” package in R, which introduces a threshold to divide the continuous exposure variable into different intervals. Associations were fitted separately on either side of the threshold to identify potential inflection points, and model fit was evaluated using the likelihood ratio test (LRT).

Finally, sensitivity analysis was performed via stratified subgroup logistic regression, and potential interactions were tested. A *p*-value > 0.05 for interaction was interpreted as no significant effect modification. All analyses were conducted using R version 4.5.1.

## Results

**Table 1** summarizes the baseline characteristics of the study cohort (n = 181) stratified by serum 25-hydroxyvitamin D [25(OH)D] levels. Compared with patients with 25(OH)D < 30 nmol/L, those with 25(OH)D ≥ 50 nmol/L had a significantly lower body mass index (BMI).

Patients with 25(OH)D levels ranging from 30 to 50 nmol/L had significantly higher admission systolic blood pressure and admission diastolic blood pressure than those with 25(OH)D < 30 nmol/L. Additionally, the duration of diabetes mellitus was significantly shorter in patients with 25(OH)D ≥ 50 nmol/L compared to those with 25(OH)D < 30 nmol/L. For lipid parameters, patients with 25(OH)D ≥ 50 nmol/L had a significantly lower total cholesterol (TC) level than those with 25(OH)D < 30 nmol/L.

A statistically significant association was observed between 25(OH)D level groups and season (*p* = 0.007). In summer, the proportion of patients with 25(OH)D ≥ 50 nmol/L was the highest (41.7%), while the proportion with 25(OH)D < 30 nmol/L was relatively low (16.2%)—this is consistent with sufficient sunlight exposure and increased vitamin D synthesis in summer (17).In spring, the proportion of patients with 25(OH)D < 30 nmol/L was the highest (27.0%), and the proportion with 25(OH)D ≥ 50 nmol/L was extremely low (2.8%), which may be attributed to insufficient vitamin D reserves following winter (18).

In **Table 2**, the incidence of lipid abnormalities across different 25(OH)D levels was compared. Among the groups, patients with 25(OH)D ≥ 50 nmol/L had a significantly lower incidence of TG abnormality than the other two groups (*p* = 0.015).

**Table 2:** Comparison of TG abnormality detection rates across different serum (25(OH)D levels.

Dyslipidemia [case (%)]	Status	<30nmol/L N=74	30~50nmol/L N=71	≥50nmol/L N=36	χ² value	*p*-value
TC	Abnormal	9(12.2)	8(11.3)	1(2.8)	2.606	0.303
	Normal	65(87.8)	63(88.7)	35(97.2)		
TG	Abnormal	25(33.8)	22(31.0)	3(8.3)^ab^	8.506	0.015
	Normal	49(66.2)	49(69.0)	33(91.7)		
HDL-C	Abnormal	51(68.9)	45(63.4)	26(72.2)	0.952	0.643
	Normal	23(31.1)	26(36.6)	10(27.8)		
LDL-C	Abnormal	6(8.1)	4(5.6)	1(2.8)	1.027	0.617
	Normal	68(91.9)	67(94.4)	35(97.2)		
non-HDL-C	Abnormal	9(12.2)	6(8.5)	1(2.8)	2.468	0.264
	Normal	65(87.8)	65(91.5)	35(97.2)		

a *p* < 0.05: compared with the 25(OH)D <30nmol/L group;

b *p* < 0.05:compared with the 25(OH)D 30-50nmol/L group. TC: Total cholesterol; TG: Triglyceride; HDL-C: High-density lipoprotein cholesterol; LDL-C: Low-density lipoprotein cholesterol; non-HDL-C: Non-high-density lipoprotein cholesterol.

As shown in **Table 3**, to evaluate the relationship between serum 25(OH)D levels and the likelihood of TG abnormality, we developed three logistic regression models with progressive adjustment for potential confounders:

**Table 3:** Multiple-adjusted logistic regression analysis of the association between 25(OH)D index and the risk of TG abnormality.

Variables	Model 1 (Univariate analysis)		Model 2		Model 3	
25(OH)D	OR(95%CI)	*p*-value	OR(95%CI)	*p*-value	OR(95%CI)	*p*-value
Age, per year	1.002(0.952~1.054)	0.943				
Gender	2.447(1.252~4.786)	0.009				
Admission systolic blood pressure	1.011(0.996~1.026)	0.148				
Admission diastolic blood pressure	1.011(0.983~1.039)	0.437				
Duration of hyperglycemia (year)	0.995(0.960~1.031)	0.767				
Season	-	0.942				
Summer	0.897(0.348~2.315)	0.823				
Autumn	0.919(0.381~2.220)	0.851				
Winter	0.757(0.311~1.840)	0.539				
25 (OH) D categorical variable	-	0.028	-	0.023		
25 (OH) D continuous variable	0.968(0.946~0.991)	0.006			0.967(0.943~0.991)	0.009

(1) Model 1 only includes the dependent variable and no covariates; (2) Model 2 includes the categorical variable of 25(OH)D, adjusted for baseline age, gender, season of 25(OH)D test, duration of diabetes, admission systolic blood pressure, and admission diastolic blood pressure; (3) Model 3 includes the continuous variable of 25(OH)D, adjusted for baseline age, gender, 25(OH)D season (set as a dummy variable), duration of hyperglycemia, admission systolic blood pressure, and admission diastolic blood pressure.

Season: Season of 25-hydroxyvitamin D test; OR: Odds Ratio; CI: Confidence Interval.

Model 1: Unadjusted, with no covariates included. Model 2: Included 25(OH)D as a categorical variable, adjusted for baseline age, gender, season of 25(OH)D measurement, duration of diabetes, admission systolic blood pressure, and admission diastolic blood pressure. Model 3: Included 25(OH)D as a continuous variable, adjusted for baseline age, gender, season of 25(OH)D measurement (set as a dummy variable), duration of diabetes, admission systolic blood pressure, and admission diastolic blood pressure.

In Model 1, for each 1 nmol/L increase in 25(OH)D, the risk of TG abnormality decreased by 3.2% (odds ratio [OR] = 0.968, 95% confidence interval [CI]: 0.946–0.991, *p* = 0.006). This negative association remained stable after adjusting for covariates in Model 3 (OR = 0.967, 95% CI: 0.943–0.991, *p* = 0.009). When modeled as a categorical variable (Model 2), higher 25(OH)D levels were also associated with a reduced risk of TG abnormality. A dose-dependent negative association between 25(OH)D levels and the risk of TG abnormality was observed across all three models.

Collinearity analysis was performed between the continuous variable of 25(OH)D] and the following variables: age, gender, duration of hyperglycemia, as well as the independent variables with *p* < 0.05 in the baseline analysis (**Table 1**) (i.e., season, admission systolic blood pressure, and admission diastolic blood pressure).The results showed that there was no collinearity between the continuous 25(OH)D variable and age, gender, duration of hyperglycemia, season, admission systolic blood pressure, or admission diastolic blood pressure. The variance inflation factor (VIF) values were 1.058, 1.172, 1.196, 1.151, 1.037, 1.801, and 1.837, respectively.

### Dose-response relationship between 25(OH)D levels and risk of TG abnormality

Restricted cubic spline (RCS) plots illustrate the dose-response relationship between 25(OH)D levels and the incidence of TG abnormality under two logistic regression models.

The solid lines represent adjusted odds ratios (OR), and the shaded bands represent 95% confidence intervals (CI). The reference value (OR = 1.0) was set at the median serum 25(OH)D level. To further examine the potential non-linear relationship between serum 25(OH)D levels and the risk of TG abnormality, RCS regression analyses were performed using the two previously defined logistic models.

**Figure 1:**
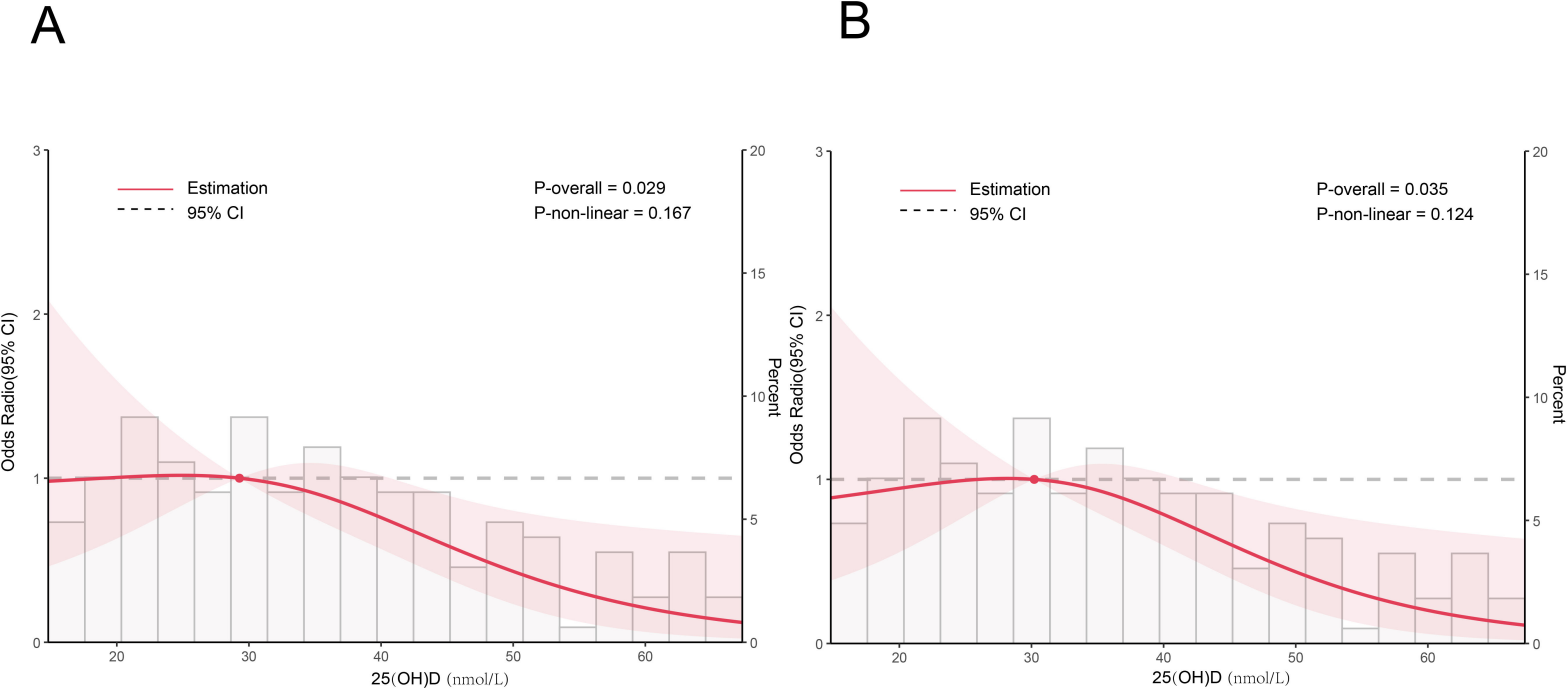
**(A)** Model 1 Unadjested; **(B)** Model 2: Adjusted for gender, age, season, systolic blood pressure (SBP), diastolic blood pressure (DBP) and duration of hyperglymcemia.

As shown in **Figure 1**, all RCS plots consistently indicated a negative association between25(OH)D levels and the odds of TG abnormality. In Model 1 (unadjusted), a distinct monotonic and approximately linear negative association was observed: the risk of TG abnormality decreased significantly at higher 25(OH)D concentrations, with a plateau at lower 25(OH)D levels. After adjusting for gender, age, season, admission systolic blood pressure, admission diastolic blood pressure, and duration of hyperglycemia, this pattern remained evident in Model 2.

Importantly, none of the models exhibited a U-shaped or J-shaped curve—this reinforces a consistent, monotonic protective effect of increased 25(OH)D levels against TG abnormality, with no significant non-linear relationship (or an approximately linear relationship) between the two. These RCS-derived results confirm the associations observed in continuous and categorical regression analyses, and further support a dose-dependent negative association, particularly when serum25(OH)D levels exceed 30.322 nmol/L.

### Threshold effect of serum 25(OH)D on the risk of TG abnormality

**Table 4** presents the results of threshold effect analysis to assess whether the association between serum 25(OH)D levels and the risk of TG abnormality follows a threshold-dependent pattern. This analysis identified 43.48 nmol/L as the inflection point derived from the data. A piecewise logistic regression model was applied, and as shown in **Table 3**, 25(OH)D was significantly associated with TG abnormality when exceeding the threshold of 43.48 nmol/L (odds ratio [OR] = 0.907, 95% confidence interval [CI]: 0.802–1.026, *p* = 0.043). However, the likelihood ratio test comparing the piecewise and standard logistic models yielded a critical p-value of 0.113, indicating a potential but not definitive improvement in model fit when allowing for a threshold effect.

**Table 4:** Threshold effect analysis of serum vitamin D_3_on TG risk using piecewise logistic regression.

Variables	Adjusted OR(95% CI)*	*p*-value
Fitting by standard logistics regression	0.969(0.947, 0.991)	0.010
Fitting by piecewise logistics regression (Breakpoint=43.48)		
VD_3_ <43.48(nmol/L)	0.998(0.955,1.044)	0.943
VD_3_ ≥43.48(nmol/L)	0.907(0.802,1.026)	0.043
Log-likelihood ratio test	-	0.113

*Adjusted for gender, age, season, duration of hyperglycemia, admission systolic blood pressure, and admission diastolic blood pressure. Season: Season of 25-hydroxyvitamin D test; OR: Odds Ratio; CI: Confidence Interval.

### Subgroup analysis and sensitivity assessment

To examine the robustness of the association between serum 25(OH)D levels and TG abnormality and potential effect modification, stratified logistic regression analyses were conducted for key subgroups.

**Figure 2:**
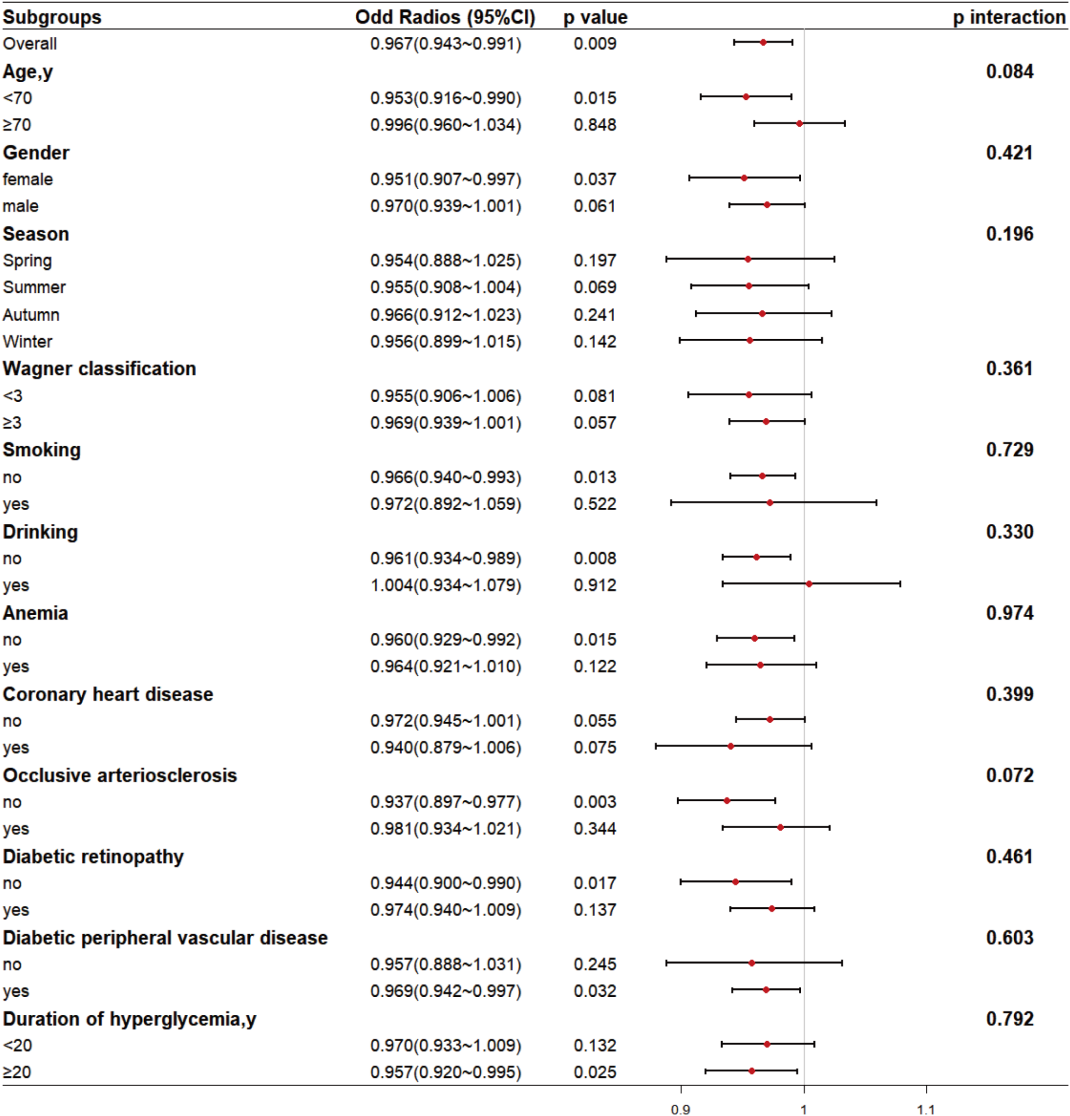
Subgroup analysis of serum vitamins D_3_ on TG Risk. Adjusted for gender, age, season, duration of hyperglycemia, admission systolic blood pressure, and admission diastolic blood pressure. Season: Season of 25 hydroxyvitamin D test; OR: Odds Ratio; CI: Confidence Interval.

As shown in **Figure 2**, after adjusting for covariates other than the stratifying variables, a significant association (P < 0.05) was observed in all covariate-stratified populations except for those stratified by season, Wagner classification, and coronary heart disease status.

Interaction terms between covariates and TG abnormality were incorporated into the models separately; results indicated no significant interaction between covariates and TG abnormality.

A statistically significant protective effect of 25(OH)D was observed in the following subgroups: Age < 70 years (OR = 0.953, 95% CI: 0.916–0.990, p = 0.015); Female gender (OR = 0.951, 95% CI: 0.907–0.997, *p* = 0.037); Non-smokers (OR = 0.966, 95% CI: 0.940–0.993, *p* = 0.013); Non-drinkers (OR = 0.961, 95% CI: 0.934–0.989, *p* = 0.008); Non-anemic (OR = 0.960, 95% CI: 0.929–0.992, *p* = 0.015); No occlusive arteriosclerosis (OR = 0.937, 95% CI: 0.897–0.977, p = 0.003); No diabetic retinopathy (OR = 0.944, 95% CI: 0.900–0.990, *p* = 0.017); With diabetic peripheral vascular disease (OR = 0.969, 95% CI: 0.942–0.997, p = 0.032); Duration of hyperglycemia ≥ 20 years (OR = 0.957, 95% CI: 0.920–0.995, *p* = 0.025).

Importantly, no significant interaction effects were observed (all p-values for interaction > 0.05), indicating that the association between VD_3_ and TG abnormality was consistent across all examined strata. The negative association between VD_3_ and TG abnormality remained consistent across all subgroups, with no evidence of significant interaction (p-values for interaction > 0.05), supporting a robust and widely applicable relationship.

## Discussion

Vitamin D deficiency has become a global health issue, and subclinical vitamin D deficiency remains prevalent worldwide, affecting up to 1 billion people in both developed and developing countries (19, 20). The endocrine functions of vitamin D include roles in the musculoskeletal system, membrane-vitamin D interaction-mediated effects, vitamin D-ionized calcium-dependent parathyroid function, and calcium-phosphate metabolism (encompassing bone mineralization as well as intestinal and renal tubular calcium absorption) (21).

Vitamin D deficiency impairs the beneficial effects of vitamin D on peripheral target cells, disrupts immune cell function, increases vulnerability to infections, exacerbates the severity of chronic diseases, and leads to higher rates of complications and premature mortality (22).

Vitamin D is first converted to 25(OH)D in the liver, and subsequently to its biologically active form, 1α,25-dihydroxyvitamin D (1α,25(OH)_2_D), in the kidneys (23). This active form binds to the vitamin D receptor (VDR) to regulate calcium and phosphate metabolism (24). Therefore, serum 25(OH)D is the most commonly measured form of vitamin D in humans, as it accurately reflects the overall vitamin D status of the body.

Previous studies have also revealed a negative correlation between age and serum 25(OH)D levels in elderly patients. This may be associated with age-related calcium loss and reduced cutaneous vitamin D synthesis, and suggests that the elderly should supplement vitamin D in a timely manner to counteract elevated parathyroid hormone (PTH) levels (25, 26).

Our study systematically investigated the association between serum 25(OH)D levels and dyslipidemia in elderly patients with diabetic foot ulcer (DFU), and found a negative association between 25(OH)D levels and triglyceride (TG) abnormality. A prospective cohort study from the UK Biobank showed that higher serum 25(OH)D concentrations were associated with a lower risk of type 2 diabetes mellitus (T2DM) across the entire glycemic spectrum below the diabetic threshold (27). Additionally, the relationship in prediabetes is modified by VDR polymorphisms, and improved triglyceride levels contribute partially to this favorable association.

Notably, a meta-analysis (28) which demonstrated that vitamin D deficiency is consistently associated with adverse lipid profiles (including elevated TG) across diverse diabetic populations. Importantly, their analysis emphasized that the interplay between vitamin D and lipids is partially mediated by chronic low-grade inflammation—a key pathological feature of diabetes—given that inflammation and dyslipidemia exhibit a mutually reinforcing relationship in diabetic patients. Our study extends this body of evidence by focusing specifically on elderly DFU patients, a high-risk subgroup where vitamin D deficiency and dyslipidemia coexist at disproportionately high rates and synergistically increase cardiovascular and ulcer-related risks. This highlights the unique clinical relevance of our findings in a population that has been historically underrepresented in vitamin D-lipid metabolism research. To our knowledge, this study is the first to validate the 25(OH)D-TG association in elderly DFU patients. While DFU patients are often complicated with neuropathy, vascular disease, and multiple metabolic disorders, our analysis showed that the inverse association between vitamin D and TG abnormality remained after adjusting for potential confounders (including age, gender, comorbidities such as hypertension). However, residual confounding factors cannot be fully ruled out—for instance, malnutrition (which frequently coexists with vitamin D deficiency and dyslipidemia in elderly patients) or unmeasured lifestyle factors may have influenced the observed association. It is also worth noting that a significant association between 25(OH)D levels and season was observed in this study (the highest proportion of high 25(OH)D levels in summer and the lowest in spring), which aligns with the physiological principle that sunlight is the primary source of vitamin D (29) and underscores the importance of accounting for seasonal variations when interpreting vitamin D-related results.

### Potential mechanisms underlying the negative association between 25(OH)D and TG abnormality

First is the metabolic regulation mechanism. By activating the vitamin D receptor (VDR), vitamin D can inhibit adipocyte differentiation, reduce the release of free fatty acids, and downregulate the expression of key enzymes involved in hepatic TG synthesis (e.g., acetyl-CoA carboxylase), thereby lowering circulating TG levels (30).

Regarding inflammation and insulin resistance: Vitamin D deficiency can exacerbate chronic low-grade inflammation (31), which is a key driver of insulin resistance and TG metabolic disorders. Conversely, adequate vitamin D may improve insulin sensitivity through anti-inflammatory effects, thereby promoting TG clearance.

Second is the vascular protective mechanism. Previous studies have shown that a single high-dose oral administration of vitamin D_2_ can improve endothelial function in patients with type 2 diabetes mellitus (T2DM) and vitamin D insufficiency (32). For patients with diabetic foot ulcer (DFU), vitamin D may reduce lipid deposition and atherosclerosis by improving vascular endothelial function, indirectly alleviating TG metabolic abnormalities caused by peripheral vascular disease.

Collectively, these mechanisms suggest that vitamin D is not only a nutrient but also likely influences the lipid profile of DFU patients through multiple pathways, including metabolic regulation, anti-inflammation, and vascular protection.

### Dose-response patterns and clinical implications

Previous clinical intervention evidence includes the following: Supplementation of 50,000 IU vitamin D for 12 weeks in DFU patients exerted beneficial effects on glucose homeostasis, total cholesterol, low-density lipoprotein cholesterol (LDL-C), total cholesterol/high-density lipoprotein cholesterol (TC/HDL-C) ratio, erythrocyte sedimentation rate (ESR), high-sensitivity C-reactive protein (hs-CRP), and malondialdehyde (MDA) levels (33); In a single-center randomized double-blind trial, high-dose vitamin D_3_(170μg) was more effective in promoting the healing of chronic diabetic foot ulcers than low-dose vitamin D_3_(20μg) (34). Two meta-analyses evaluating the reliability of vitamin D supplementation on clinical outcomes of diabetic foot ulcers demonstrated that vitamin D supplementation, as an adjunctive therapy for DFU, is beneficial (15, 35).

The results of this study showed that restricted cubic spline (RCS) analysis revealed an approximately linear negative correlation between 25-hydroxyvitamin D (25(OH)D) levels and triglyceride (TG) abnormality, with no obvious clinically meaningful inflection point identified. Although threshold effect analysis suggested that 43.48 nmol/L might be a potential statistical inflection point, since it is a result derived from data-driven analysis, it can be defined as an exploratory finding rather than a confirmed threshold with clinical guiding significance.

Our study has several strengths. It focuses on elderly DFU patients—a high-risk population that has been understudied—and fills the evidence gap regarding the association between vitamin D and lipid metabolism. We employed multiple model adjustments (including stratified analysis and continuous variable analysis), restricted cubic spline (RCS) analysis, and threshold effect analysis to systematically verify the stability of the observed association. Additionally, subgroup analyses covered key factors such as age, gender, and comorbidities, which confirms the robustness of the results.

Our study also has some limitations. The retrospective design precludes establishing a causal relationship, so prospective intervention studies are needed to verify the impact of vitamin D supplementation on triglyceride (TG) levels and DFU prognosis. The single-center setting and limited sample size may restrict the generalizability of the results. Moreover, we did not include information on vitamin D supplementation dosage and duration, making it impossible to quantify the intervention effect. Furthermore, some potential confounding factors (such as dietary structure and exercise habits) were not fully controlled, which may affect the interpretation of the results.

## Conclusion

This study found that in diabetic foot ulcer (DFU) patients aged ≥ 60 years, serum 25-hydroxyvitamin D (25(OH)D) levels exhibit a dose-dependent negative association with triglyceride (TG) abnormality. However, due to the observational cross-sectional design of this study, further investigation via prospective or interventional trials is needed in the future. In the clinical practice for elderly DFU patients, assessing vitamin D status (e.g., maintaining adequate levels through sunlight exposure or supplements) can be considered a preliminary measure. Future multicenter randomized controlled trials are required to determine the optimal dosage and timing of vitamin D supplementation, thereby providing higher-level evidence for the precise management of diabetic foot ulcers.

## Data Availability

The original contributions presented in the study are included in the article/supplementary material. Further inquiries can be directed to the corresponding author.
